# Hemostatic parameters in transgender women receiving gender-affirming hormone therapy: A shift to a cisgender female pattern?

**DOI:** 10.1371/journal.pone.0323606

**Published:** 2025-05-14

**Authors:** Paloma Dias da Cruz, Betânia Rodrigues Santos, Poli Mara Spritzer

**Affiliations:** 1 Gynecological Endocrinology Unit, Division of Endocrinology, Hospital de Clínicas de Porto Alegre (HCPA), Porto Alegre, Rio Grande do Sul (RS), Brazil; 2 Post-Graduate Program in Endocrinology, Medicine School, Universidade Federal do Rio Grande do Sul (UFRGS), Porto Alegre, RS, Brazil; 3 Department of Physiology and Post-Graduate Program in Physiology, UFRGS, Porto Alegre, RS, Brazil; Federal University of Minas Gerais: Universidade Federal de Minas Gerais, BRAZIL

## Abstract

Transgender women have an increased incidence of venous thromboembolism (VTE) compared with cisgender individuals. However, data on hemostatic parameters in this population are scarce. We aimed to evaluate hemostatic parameters in transgender women receiving gender-affirming hormone therapy (GAHT) compared with cisgender controls. We conducted a cross-sectional study including 40 transgender women (sample size based on prior calculation), and age- and body mass index-matched cisgender women (n = 25) and cisgender men (n = 25) as controls. Blood samples were collected between 2016 and 2023. We assessed hemostatic parameters (plasminogen activator inhibitor-1 [PAI-1], free protein S, vascular cell adhesion molecule-1, antithrombin, anticoagulant protein C, prothrombin time activity, thrombin time), hormonal profile (estradiol, sex hormone-binding globulin, estrogen dose, total testosterone, and free androgen index), and inflammatory markers (fibrinogen, C-reactive protein, and leukocyte count). Transgender women (mean [SD] age, 30.6 [8.0] years; median GAHT duration, 36.5 months) and cisgender women had similar hemostatic and inflammatory parameters. Compared with cisgender men, transgender women had higher PAI-1 levels (p = 0.001) and lower free protein S levels (p = 0.023). No differences were found in other hemostatic parameters between the groups. In conclusion, transgender women on long-term GAHT had higher levels of PAI-1 and lower levels of free protein S than cisgender men, indicating a slightly more prothrombotic profile. However, their hemostatic and inflammatory parameters were similar to those of cisgender women, suggesting a shift towards a female pattern. Factors beyond GAHT may contribute to the increased risk of VTE in this population.

## Introduction

Gender-affirming hormone therapy (GAHT) is a treatment often sought by transgender people that aims to align physical characteristics with gender identity. The use of GAHT has been associated with an increase in quality of life, and a decrease in depression and anxiety [1,2]. In transgender women, a standard GAHT regimen generally consists of a combination of a natural estrogen with an antiandrogen or a gonadotropin-releasing hormone agonist. The use of synthetic estrogen ethinyl estradiol (EE) is contraindicated due to increased risk of venous thromboembolism (VTE) [3,4].

The long-term risks of GAHT are still under investigation, particularly its hemostatic effects. Changes in clinical practice reflect the attention to reducing the risk of venous thromboembolism (VTE), such as the sharp reduction in EE prescriptions [3,4] and, perhaps, the use of lower doses of estrogens and cyproterone acetate (CPA) [5].

However, recent retrospective observational studies have demonstrated increased VTE risk in transgender women compared with cisgender men and women, even after removing individuals using EE [6–8]. A recent meta-analysis confirmed the higher prevalence of VTE in transgender women and the association with older age and longer use of estrogen [9]. Higher rates of VTE have also been reported in transgender women taking GAHT compared to transgender men [10].

Changes in hemostatic parameters have been implicated in VTE risk. Estrogen-containing hormone therapy (HT) such as oral contraceptives and menopausal HT are well-known VTE risk factors and induce predominantly procoagulant changes [11,12]. Indirect comparisons in studies with cisgender women suggest no significant difference in HT effects on hemostasis between non-synthetic oral estrogens [12], but there is variability in the evidence to date [13]. Regarding transdermal 17β-estradiol, multiple studies of postmenopausal women have consistently shown that it has little or no effect on markers of coagulation and fibrinolysis [11], as well as on the incidence of VTE [12–15].

However, data on the risk of VTE and hemostatic alterations in cisgender women undergoing HT cannot be interpreted as the same as data from transgender women on GAHT, due to differences in estrogen doses, the associated use of antiandrogens, and sexual dimorphism [9].

Few studies have assessed the effect of different GAHT regimens on hemostatic parameters in transgender women, often comparing baseline to 2–12 months of GAHT but without a control group. To date, increased levels of procoagulant factors have been detected [16–19], as well as altered levels of anticoagulant proteins [18]. A decrease in fibrinolytic pathway proteins has also been demonstrated with the use of EE [20] but not with transdermal estradiol [16,19,21]. A transient increase has been observed in inﬂammatory cytokines such as interleukin (IL)-6, and IL-8 [18] with the use of conjugated equine estrogens, while a decrease has been noted in systemic and endothelial inflammatory markers (high-sensitivity C-reactive protein [hs-CRP] and vascular cell adhesion molecule-1 [VCAM-1]) with the use of transdermal estradiol [19].

The present study aimed to evaluate hemostatic parameters in transgender women receiving GAHT compared with cisgender control groups and to determine whether these parameters are associated with inflammatory markers or hormonal factors. The study also seeks to contribute indirect insights into the safety of GAHT use.

## Materials and methods

### Participants and study protocol

This is a cross-sectional study of transgender women followed up at the endocrine outpatient clinic of the Transdisciplinary Gender Identity Program of Hospital de Clínicas de Porto Alegre, Brazil. We included participants who had been prospectively recruited by our research group and had serum samples stored at −80°C in a biorepository from previous studies [2,22]. The aliquots were collected at a single time point for each participant between 2016 and 2023.

The control groups consisted of age- and body mass index (BMI)-matched cisgender women and cisgender men with no comorbidities and no use of medications that could interfere with coagulation parameters who were recruited from advertisement on the hospital’s home page. The same collections for laboratory tests and for biorepository were performed for controls. Blood samples from cisgender women were obtained during the follicular phase of the menstrual cycle.

Transgender women aged ≥16 years on GAHT were considered eligible if serum and plasma had been collected and stored during regular use of GAHT, according to a review of their medical records. Those who had undergone gender-affirming surgery with gonadectomy were also included.

Participants were excluded if they were not using a standard GAHT regimen, had undergone a surgical procedure up to 2 months before blood collection, or had hs-CRP levels >10 mg/L. None of the participants were on antiplatelet or anticoagulant therapy.

GAHT consisted of non-synthetic estrogens combined with an antiandrogen. The treatment doses were individualized according to clinical response. Antiandrogens were discontinued in participants who had undergone gonadectomy. Demographic and clinical data from all participants were obtained through a review of electronic medical records.

The study protocol was approved by the Research Ethics Committee of Hospital de Clinicas de Porto Alegre and complies with the Declaration of Helsinki. Written informed consent was obtained from all participants during the recruitment process. All participant data were de-identified before analysis.

### Laboratory analysis and assays

Venous blood samples were collected between 8–10 AM after a 12-hour overnight fast. The hs-CRP was determined by immunoturbidimetry (Alinity c, Abbott Laboratories). Prothrombin time (PT) activity and fibrinogen were determined by coagulometry (Sta R Max, Stago). Thrombin time and antithrombin were measured by a coagulometric method (Sta R Compact, Stago), but in 2023 antithrombin was measured by a chromogenic method (ACL TOP550, Werfen) and subjected to a quantitative validation. Data on thrombin time were analyzed in a subsample of the participants included up to 2022 (n = 65), since it was only possible to perform a qualitative validation between the methods. Anticoagulant protein C activity was measured by a chromogenic method (ACL TOP300, Werfen). Free protein S was measured by immunoturbidimetry (Sta R Compact, Stago), with intra-assay and inter-assay coefficients of variation (CVs) ≤3.0%. All other intra-assay and inter-assay CVs were ≤10.0%.

Magnetic bead-based immunoassay of PAI-1 (PAI-1 Human ProcartaPlex Simplex Kit, Invitrogen, ThermoFisher, Luminex 100/200 System; assay range, 0.026–107.2 ng/mL) and VCAM-1 measurements (VCAM-1 Human ProcartaPlex Simplex Kit, Invitrogen, ThermoFisher; assay range, 0.012–47 ng/mL) were performed on the same day using kits of the same lot.

Estradiol was measured by electrochemiluminescence immunoassay (Cobas e602, Roche Diagnostics), sex hormone-binding globulin (SHBG, Immulite2000, Siemens) and total testosterone levels were measured by chemiluminescence immunoassay (Advia Centaur XP, Siemens). Free androgen index (FAI) was calculated by dividing total testosterone (nmol/L) by SHBG (nmol/L) x100.

### Sample size calculation

Sample size was calculated using the PSS Health tool, version 0.4.0, based on data of a previous study [19], to detect a mean difference of 7.7IU/dL in the natural anticoagulant protein C levels between assessments after 12 months of GAHT. Based on data from this previous study, a sample size of 22 participants per group was required for a power of 80% and a significance level of 5%. Adding 10% for possible losses, the calculated sample size was 25 participants per group.

### Statistical analysis

Normality of data distribution was assessed using the Shapiro-Wilk test. Data are expressed as mean and standard deviation for variables with normal distribution and as median and interquartile range for variables with non-Gaussian distribution. Non-Gaussian data were log-transformed before statistical analysis and then back-transformed to their original scale for data reporting. Categorical variables are expressed as absolute numbers and percentages. The groups were compared by one-way analysis of variance, followed by Tukey’s post hoc test for continuous variables and by Fisher’s exact test for categorical variables. Data were analyzed with SPSS for Windows, version 18.0 (SPSS Inc., Chicago, IL, USA). A p-value<0.05 was considered statistically significant.

## Results

**Fig 1:**
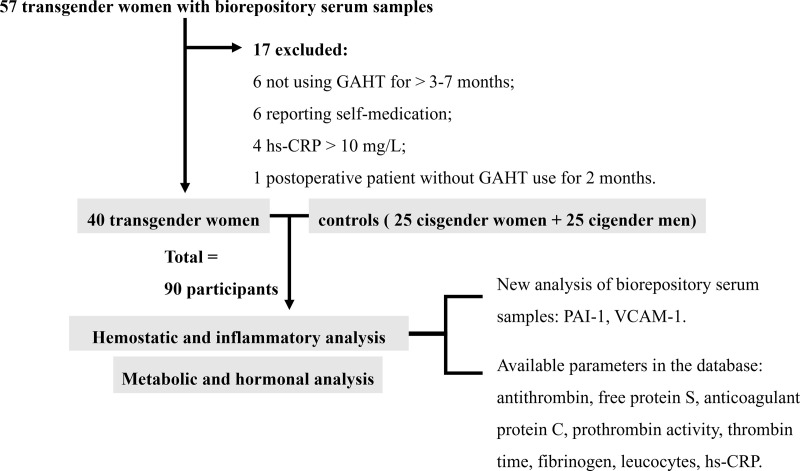
Flowchart of inclusion and exclusion criteria for participants and sample analysis.

Of a total of 57 transgender women with serum samples in the biorepository, 17 were excluded: 6 for not using GAHT for > 3–7 months at the time of collection; 6 for reporting self-medication with no previous outpatient medical visit; 4 with hs-CRP > 10 mg/L; and 1 postoperative participant who discontinued GAHT for 2 months (Fig 1).

The study included 40 transgender women aged 17–49 years receiving GAHT for a median (IQR) of 36.5 (30.5–55.7) months. Of these, 6 meet criteria for prediabetes, 1 had type 2 diabetes mellitus (treated with metformin and with glycemia within goal), 3 were on selective serotonin reuptake inhibitors, and 6 were living with HIV on antiretroviral therapy and had undetectable viral load. In addition, 50 age- and BMI-matched cisgender controls (25 cisgender women and 25 cisgender men) were selected. No participant had known atherosclerotic disease, renal or hepatic insufficiency, current malignancy, or a personal or family history of VTE.

Clinical, hormonal, and metabolic features of participants are summarized in Table 1. The groups did not differ in terms of age, BMI, prevalence of overweight and obesity, or smoking status. Systolic and diastolic blood pressure were significantly higher in transgender women than in cisgender women but did not differ from those in cisgender men. Although only the transgender group had individuals diagnosed with prediabetes and diabetes mellitus, fasting glucose did not differ between the 3 groups. Lipid profile was similar between groups and into the reference values, except for HDL-cholesterol that was slight but significantly higher in cisgender women than in transgender women and cisgender men (p = 0.027) (S1 Table).

**Table 1:** Clinical, metabolic, and hormonal features of transgender women and cisgender controls at the time of sample collection.

Variable	Transgender women (n = 40)	Cisgender women (n = 25)	Cisgender men (n = 25)	p
Age, years	30.6 ± 8.0	30.9 ± 6.2	29.5 ± 6.4	0.757
BMI, kg/m²	25.6 ± 4.5	25.2 ± 4.8	25.5 ± 3.5	0.938
Overweight, n (%)	10 (25)	9 (36)	11 (44)	0.573
Obesity, n (%)	8 (20)	3 (12)	3 (12)	0.573
Current smoking, n (%)	2 (5)	2 (8)	0 (0)	0.655
Former smoking, n (%)	4 (10)	2 (8)	1 (4)	0.655
SBP, mm Hg	120 (110–123)^a^	100 (100–110)^b^	110 (110–120)^a^	< 0.001
DBP, mm Hg	80 (70–80)^a^	75 (60–80)^b^	80 (70–80)^a^	0.018
Glucose, mg/dL	87 (82 - 94)	85 (83 - 88)	88 (84 - 93)	0.089
Estradiol, pg/mL^1^	46.1 (32.8–56.5)^a^	72.1 (44.4–119.1)^b^	20.9 (15.6–26.9)^c^	< 0.001
SHBG, nmol/L	60.2 (35.3–129.2)^a^	59.9 (37.4–73.9)^a^	27.9 (20.8–34.7)^b^	< 0.001
TT, ng/mL	0.18 (0.11–1.04)^a^	0.28 (0.19–0.38)^a^	5.10 (3.71–5.68)^b^	< 0.001
FAI	0.96 (0.26–6.40)^a^	1.71 (1.12–2.75)^a^	57.58 (50.55–68.89)^b^	< 0.001

Values are expressed as mean (standard deviation) or median (interquartile range) (one-way analysis of variance, Tukey’s post hoc test) or absolute numbers and percentages (Fisher’s exact test). Different superscript letters in the same row indicate statistically significant differences. BMI: body mass index; Overweight: BMI of 25 to 29.9 kg/m^2^; Obesity: BMI ≥ 30 kg/m^2^; SBP: systolic blood pressure; DBP: diastolic blood pressure; SHBG: sex hormone-binding globulin; TT: Total testosterone; FAI: free androgen index; ^1^ n = 85.

Regarding hormonal profile, transgender women had median estradiol levels lower than those of cisgender women but higher than those of cisgender men. Transgender women and cisgender women had similar SHBG, total testosterone, and FAI levels, and both groups had statistically higher SHBG (p < 0.001) and statistically lower testosterone and FAI (p < 0.001) than cisgender men.

The GAHT regimens used by transgender women are described in Table 2. Most participants (65.0%) were using oral estradiol valerate, at a mean daily dose of 2.7mg, and spironolactone as an antiandrogen (52.5%). Some individuals were not using antiandrogens (32.5%), most of whom had undergone gonadectomy.

**Table 2:** Gender-affirming hormone therapy in transgender women (n = 40).

Variable	n (%)	Mean daily dose, mg (minimum - maximum)
**Estrogen formulations:**		
Oral estradiol valerate	26 (65.0)	2.7 (1 - 4)
Oral CEE	11 (27.5)	1.4 (0.625 - 1.875)
Transdermal estradiol gel	3 (7.5)	1.2 (1.0 - 1.5)
**Antiandrogens:**		
Spironolactone	21 (52.5)	83.3 (50 - 150)
Cyproterone	6 (15.0)	45.8 (25 - 100)
No use	13 (32.5)	
**Underwent gonadectomy**	11 (27.5)	
**GAHT duration, months**	36.5 (30.5–55.7)^a^	

CEE: conjugated equine estrogens; GAHT: gender-affirming hormone therapy; ^a^ median (interquartile range).

**Fig 2:**
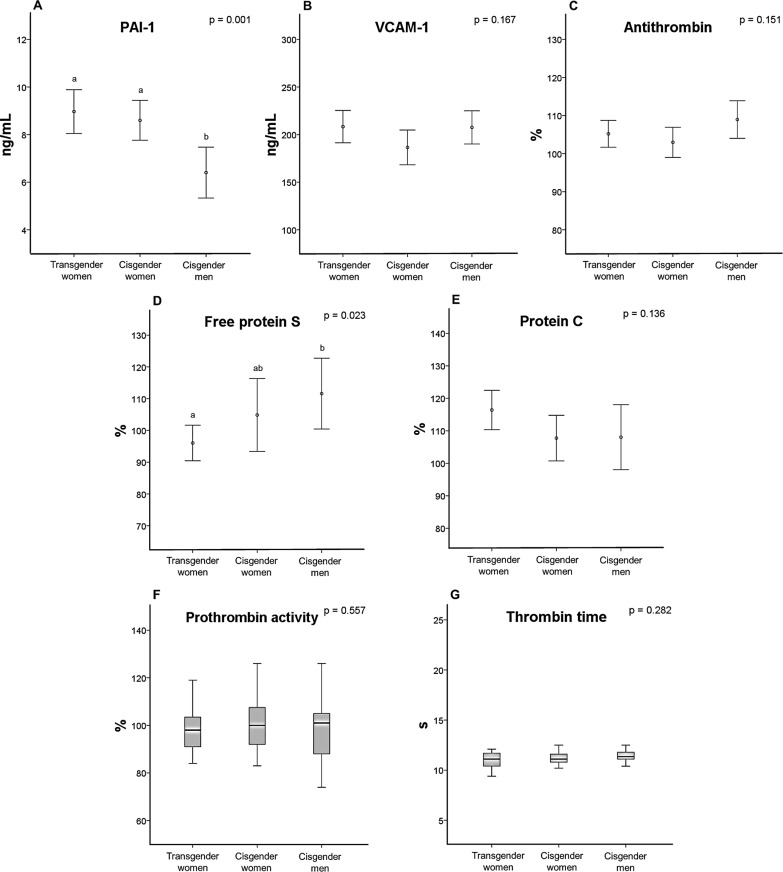
Hemostatic parameters in transgender women and cisgender controls (panels A-G). Different letters indicate statistically significant differences (one-way analysis of variance, Tukey’s post hoc test). PAI-1: plasminogen activator inhibitor-1; VCAM-1: vascular cell adhesion molecule-1.

Fig 2 and S2 Table show the results for hemostatic parameters in the 3 groups. Transgender women had PAI-1 levels higher than those of cisgender men but comparable to those of cisgender women (p = 0.001) (Fig 2A). Free protein S levels differed between the groups (p = 0.023), with lower levels in transgender women than in cisgender men (Fig 2D). All other hemostatic parameters evaluated (Fig 2 panels B,C,E-G) did not differ between the 3 groups.

**Fig 3:**
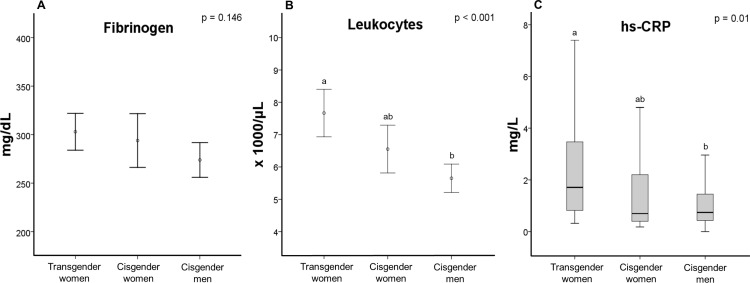
Inflammatory parameters in transgender women and cisgender controls (panels A-C). Different letters indicate statistically significant differences (one-way analysis of variance, Tukey’s post hoc test). hs-CRP: high-sensitivity C-reactive protein.

Fig 3 and S2 Table show the results for inflammatory parameters in the 3 groups. Leukocyte count and hs-CRP levels (Fig 3B and C) were significantly higher in transgender women than in cisgender men (p < 0.001 and p = 0.013, respectively), but these parameters did not differ between cisgender women and the other groups. Mean fibrinogen levels were similar across the groups (p = 0.146) (Fig 3A).

A sensitivity analysis removing the 3 participants receiving transdermal GAHT showed that the between-group differences in inflammatory and hemostatic parameters persisted. The same occurred after removing the participants with prediabetes and diabetes mellitus from the analyses.

## Discussion

In the present study, transgender women using GAHT for a median of 36.5 months had hemostatic and inflammatory parameters similar to those of cisgender women. To our knowledge, this study is the first to compare PAI-1 and free protein S with data from a control group of cisgender women. Compared with cisgender men, transgender women had higher levels of PAI-1 and lower levels of free protein S, indicating a slightly more prothrombotic profile, but still comparable to that of cisgender women. These findings offer some reassurance regarding the risk of standard GAHT-related hemostatic changes in this population.

Our choice of study parameters was based on their implication in VTE risk. Elevated PAI-1 levels appear to increase the risk of atherothrombotic events and may also promote the progression of vascular disease [23,24], while lower anticoagulant protein C, free protein S and antithrombin (natural anticoagulants) are associated with risk of future thrombosis [25,26]. Plasma VCAM-1 levels are associated with risk of atherosclerotic disease, but they may also have a potential role in mediating acute venous thrombosis [27,28].

In studies comparing cisgender women before and after starting HT, PAI-1 has been consistently found to decrease with the use of oral contraceptives and menopausal HT [29–33]. Data from transgender women using transdermal estradiol and CPA for 4–12 months did not show differences in PAI-1 concentrations [19,21], but a reduction in PAI-1 levels was observed in a group using oral EE 100 mcg per day [21], pointing out differences according to estrogen formulation and route of administration.

The only published study, to our knowledge, of transgender women compared with both cisgender women and cisgender men as controls [34] found a hypercoagulability profile accompanied by an increase in the overall fibrinolytic potential, with a shift towards the parameters of cisgender women with no evident difference between users of natural oral or transdermal estrogen. Decreased PAI-1 levels may be a surrogate for enhanced fibrinolytic activity. In the present study, although significantly lower PAI-1 levels were found in cisgender men, the mean values were within the reference range in all 3 groups and no participant had levels above the reference value.

Our results are consistent with those of Lake et al [35], who reported that transgender women have higher IL-8 and PAI-1 than cisgender men, but no comparison with cisgender women was performed. Their findings were independent of HIV serostatus, but self-reported use of GAHT was unfortunately incomplete and serum estradiol and testosterone concentrations were not available. Nevertheless, the fact that one-third of transgender women were not on GAHT but had biomarker changes like those on GAHT suggests the likely presence of other mediating factors.

In this context, it is important to point out the relationship between PAI-1 and inflammation. Indeed, we identified increased hs-CRP in transgender women compared with cisgender men, but not compared with cisgender women. PAI-1 synthesis is directly influenced by circulating levels of inflammatory cytokines, which has adipocytes as a major source. PAI-1 levels are consistently correlated with insulin resistance and type 2 diabetes mellitus [36] and, in the present study, we cannot rule out the hypothesis that the PAI-1 concentrations found in transgender women are partially related to proinflammatory status and metabolic dysfunction. Yet, our study groups were matched for BMI and the results found in transgender women were not different from those in cisgender women.

An increase in hepatic synthesis of hs-CRP has been linked to menopausal HT [33,37]. Furthermore, the route of administration appears to impact: oral administration of estrogen has been observed to elevate hs-CRP, whereas transdermal administration seems to have no discernible effect on circulating hs-CRP levels [33,38]. In this sense, as transgender subjects are the only group using exogenous estrogen, and most of them orally (92.5%), the higher hs-CRP levels found may be more related to increased liver synthesis than to inflammation per se.

It is known that free protein S varies according to sex, age, and hormonal status [39], with lower levels in cisgender women than in cisgender men, particularly at a young age. The levels found in transgender women in our study were similar to those in cisgender women but lower than those in cisgender men, suggesting a shift to a cisgender female profile. Furthermore, oral estrogen use is known to reduce protein S levels in cisgender women, but a significant dose effect has not been clearly demonstrated [11,39].

It is notable that the prevalence of smoking was lower than that reported in previous studies in the transgender population [6,7,18,35], and it did not differ from that in the cisgender control groups. Although we did not exclude transgender women with comorbidities, sensitivity analyses showed no influence of prediabetes, or diabetes mellitus on the differences found in free protein S and PAI-1.

Serum estradiol concentrations observed in transgender women were lower than those in cisgender women, but within the reference range for the follicular phase, and higher than those in cisgender men. Despite this, SHBG and testosterone concentrations in transgender women were similar to those in cisgender women. Oral estradiol is metabolized via the hepatic first-pass effect and increases SHBG production in a dose-dependent manner [40]. GAHT-induced increase in SHBG levels is associated with reduced testosterone bioavailability, resulting in promotion of feminization. Moreover, SHBG levels in our study were negatively associated with free protein S, which is in line with previous studies [19,41].

Our findings are based on medically prescribed and supervised GAHT. However, transgender people may still face barriers to accessing healthcare. Although 17β-estradiol and estradiol valerate are the current standard of care, EE is unfortunately still used in self-medication practices – often at high doses. In this scenario, one would expect greater changes in the parameters assessed.

Previous studies have identified an increased VTE risk in transgender women, when using cisgender women as a comparator [6,7]. The changes in hemostatic and inflammatory markers observed in the current study, however, reflect a pattern similar to that of the identity gender. It is believed that factors other than GAHT are involved in atherothrombotic risk. Indeed, a large retrospective cohort study showed an increased mortality risk in transgender women, but this increase was mainly explained by causes of death not associated with GAHT [42]. The transgender population still faces multiple social, economic, and health disparities, and contributors to general and minority stressors can negatively impact health outcomes [43].

As a strength of this study, well-matched control groups of both cisgender women and cisgender men were included. This design allowed the comparison between groups at a time when GAHT had been used for a longer period. Our study provides real-world evidence of transgender women on GAHT for a median of 36.5 months, a period longer than that reported in all studies published to date evaluating hemostatic variables. Furthermore, we investigated a selection of hemostatic parameters with documented changes in cisgender women on HT which are associated with VTE risk. Limitations of this study include the cross-sectional design, precluding conclusions about the direction of causality. The study has a small sample size and although it was adequate according to the sample size calculation, we could not assess the impact of different GAHT regimes, including differences in doses, routes, and antiandrogens. It was not possible to assess the hemostatic parameters in older women, who are more likely to have a thrombotic event [9].

## Conclusion

In conclusion, transgender women using medically prescribed GAHT for a long-term period had hemostatic and inflammatory parameters similar to those of cisgender women not on HT. Compared with cisgender men, transgender women had higher levels of PAI-1 and lower levels of free protein S, indicating a slightly more prothrombotic profile, but still comparable to that of cisgender women. The findings of our study provide some reassurance about the risk of GAHT-related hemostatic changes in transgender women. It is possible that unsupervised therapy and factors other than GAHT use are involved in the increased risk of VTE. Further research is needed, including prospective studies of transgender women on long-term GAHT.

## Supporting information

S1 Table Lipid profile of transgender women and cisgender controls. Values are expressed as mean ± standard deviation or median (interquartile range) (one-way analysis of variance, Tukey’s post hoc test). Different superscript letters in the same row indicate statistically significant differences. HDL: high density cholesterol; LDL: low density cholesterol.
(DOCX)

S2 Table Hemostatic and inflammatory parameters of transgender women and cisgender controls. Values are expressed as mean ± standard deviation or median (interquartile range) (one-way analysis of variance, Tukey’s post hoc test). Different superscript letters in the same row indicate statistically significant differences. PAI-1: plasminogen activator inhibitor-1; VCAM-1: vascular cell adhesion molecule-1; hs-CRP: high-sensitivity C-reactive protein; ^1^ n = 65.
(DOCX)

S1 Data (PDF)
