# Supplementary material for: Hemostatic parameters in transgender women receiving gender-affirming hormone therapy: A shift to a cisgender female pattern?
Source: PLoS One. 2025 May 14;20(5):e0323606. doi: 10.1371/journal.pone.0323606 (PMC12077691; doi:10.1371/journal.pone.0323606)
Supplement: S1 Table — Values are expressed as mean ± standard deviation or median (interquartile range) (one-way analysis of variance, Tukey’s post hoc test). Different superscript letters in the same row indicate statistically significant differences. HDL: high density cholesterol; LDL: low density cholesterol. (DOCX) [file pone.0323606.s001.docx]

**S1 Table. Lipid profile of transgender women and cisgender controls.**

| **Variables** | **Transgender women**  **(40)** | **Cisgender women**  **(25)** | **Cisgender men**  **(25)** | **p** |
| --- | --- | --- | --- | --- |
| Total cholesterol, mg/dL | 171 (147 - 208) | 181 (162 - 202) | 166 (148 - 200) | 0.314 |
| HDL, mg/dL | 50.9 ± 13.4 ^a^ | 60.3 ± 20.5 ^b^ | 50.2 ± 10.0 ^ab^ | **0.027** |
| LDL, mg/dL | 100.7 ± 30.7 | 106.7 ± 29.5 | 99.0 ± 29.8 | 0.631 |
| Triglycerides, mg/dL | 97.0 (72.3 - 128.8) | 74.0 (51.5 - 102.5) | 81.0 (55.0 - 137.0) | 0.325 |

Values are expressed as mean ± standard deviation or median (interquartile range) (one-way analysis of variance, Tukey’s post hoc test). Different superscript letters in the same row indicate statistically significant differences. Bold p-values indicate statistical significance at the p < 0.05 level. HDL: high density cholesterol; LDL: low density cholesterol.
