# Supplementary material for: Hemostatic parameters in transgender women receiving gender-affirming hormone therapy: A shift to a cisgender female pattern?
Source: PLoS One. 2025 May 14;20(5):e0323606. doi: 10.1371/journal.pone.0323606 (PMC12077691; doi:10.1371/journal.pone.0323606)
Supplement: S2 Table — Values are expressed as mean ± standard deviation or median (interquartile range) (one-way analysis of variance, Tukey’s post hoc test). Different superscript letters in the same row indicate statistically significant differences. PAI-1: plasminogen activator inhibitor-1; VCAM-1: vascular cell adhesion molecule-1; hs-CRP: high-sensitivity C-reactive protein; 1 n = 65. (DOCX) [file pone.0323606.s002.docx]

**S2 Table. Hemostatic and inflammatory parameters of transgender women and cisgender controls.**

| **Variable** | **Transgender women (n = 40)** | **Cisgender women (n = 25)** | **Cisgender men**  **(n = 25)** | **p** |
| --- | --- | --- | --- | --- |
| PAI-1, ng/mL | 8.97 ± 2.86 ^a^ | 8.60 ± 2.03 ^a^ | 6.40 ± 2.59 ^b^ | **0.001** |
| VCAM-1, ng/mL | 208.39 ± 53.14 | 186.54 ± 44.30 | 207.59 ± 42.16 | 0.167 |
| Antithrombin, % | 105.22 ± 11.02 | 102.98 ± 9.61 | 108.96 ± 11.94 | 0.151 |
| Free protein S, % | 96.04 ± 16.52 ^a^ | 104.85 ± 22.32 ^ab^ | 111.59 ± 25.14 ^b^ | **0.023** |
| Anticoagulant Protein C, % | 116.40 ± 18.96 | 107.75 ±16.65 | 108.04 ± 23.11 | 0.136 |
| Prothrombin activity, % | 98.00 (91.00 - 104.00) | 100.00 (92.00 - 108.75) | 101.00 (87.50 - 106.00) | 0.557 |
| Thrombin time, s^1^ | 11.10 (10.40 - 11.73) | 11.10 (10.75 - 11.65) | 11.35 (11.08 - 11.88) | 0.282 |
| Fibrinogen, mg/dL | 302.95 ± 58.55 | 293.92 ± 67.17 | 273.88 ± 43.39 | 0.146 |
| Leukocytes, x10^3^/µL | 7.67 ± 2.29 ^a^ | 6.55 ± 1.79 ^ab^ | 5.65 ± 1.06 ^b^ | **< 0.001** |
| hs-CRP, mg/L | 1.71 (0.81 - 3.50) ^a^ | 0.70 (0.40 - 2.63) ^ab^ | 0.74 (0.42 - 1.54) ^b^ | **0.010** |

Values are expressed as mean ± standard deviation or median (interquartile range) (one-way analysis of variance, Tukey’s post hoc test). Different superscript letters in the same row indicate statistically significant differences. Bold p-values indicate statistical significance at the p < 0.05 level. PAI-1: plasminogen activator inhibitor-1; VCAM-1: vascular cell adhesion molecule-1; hs-CRP: high-sensitivity C-reactive protein; ^1^ n = 65.
