# Supplementary material for: Hemostatic parameters in transgender women receiving gender-affirming hormone therapy: A shift to a cisgender female pattern?
Source: PLoS One. 2025 May 14;20(5):e0323606. doi: 10.1371/journal.pone.0323606 (PMC12077691; doi:10.1371/journal.pone.0323606)
Supplement: S1 Data — (PDF) [file pone.0323606.s003.pdf]

ID	Group	Age, years	BMI, kg/m²	SBP, mmHg	DBP, mmHg	Glucose, mg/dL	Estradiol, pg/mL	SHBG, nmol/L	TT, ng/mL	PAI-1, ng/mL	VCAM-1, ng/mL	Antithrombin, %	Free protein S, %
1	Trans women	46	28.04	999	999	90	27.9	44.7	8.42	8.989	263.28	111	106.7
2	Trans women	28	33.56	120	80	85	479.6	63.6	0.22	10.397	210.85	115.3	115
3	Trans women	29	25.04	120	80	87	999	145.7	0.26	9.038	252.19	106	999
4	Trans women	35	27.05	110	80	82	999	167.7	0.15	14.015	195.58	94.2	100.8
5	Trans women	20	23.86	110	70	87	77.3	40.1	2.29	9.327	177.39	109.9	95.1
6	Trans women	30	30.4	130	80	77	101.7	83.3	0.17	7.091	191.76	113.6	120
7	Trans women	34	22.21	130	90	93	43.7	70.7	6.43	8.316	251.16	108.9	68.9
8	Trans women	26	35.89	120	80	89	32.8	32.8	3.7	6.182	337.53	103.4	87.902
9	Trans women	27	21.44	110	80	83	999	175.1	0.025	11.365	277.19	105.3	75.515
10	Trans women	22	18.17	100	70	84	9.9	34.7	3.4	10.961	231.08	110.8	101.685
11	Trans women	47	23.47	130	80	112	35.4	125.6	0.06	5.586	130.2	106	99.4
12	Trans women	41	32.65	140	100	92	37.6	37.1	0.03	4.614	295.2	108.3	136.7
14	Trans women	33	22.39	110	70	110	999	43.9	0.09	5.708	181.92	106.2	94.286
16	Trans women	18	21.51	999	999	95	31.1	130.4	0.11	5.277	286.53	119.3	68.007
17	Trans women	33	22.99	999	999	82	42.9	50.4	0.06	10.544	190.72	122.3	96.1
18	Trans women	27	21.8	110	70	91	49	73.2	1.19	11.275	215.27	97.6	109.298
19	Trans women	36	23.67	120	70	103	108	234	0.08	6.884	242.02	67.1	74.476
20	Trans women	46	23.88	120	80	111	42.1	38	0.025	10.626	228.29	114.8	97.7
21	Trans women	49	30.93	130	80	103	56.5	29.5	0.21	9.383	217.73	113.3	97.104
22	Trans women	17	18.94	120	80	87	48.3	25.9	4.81	1.005	180.84	113.6	115.6
23	Trans women	35	22.72	120	80	95	12	162.3	0.11	6.863	178.32	86.3	88.94
24	Trans women	25	24.31	110	70	79	99	74.1	10.72	9.698	128.26	112.5	103.8
25	Trans women	20	24.91	110	70	81	999	216.7	0.11	11.933	159.91	114	59.8
27	Trans women	31	29.35	130	80	86	46.1	43	0.025	9.605	308.47	102.9	87.7
28	Trans women	41	30.67	130	100	99	42.5	124.2	0.025	11.583	280.54	123.2	83.507
31	Trans women	32	30.37	110	70	80	40.1	69.6	0.11	11.443	145.02	92.8	106.658
32	Trans women	27	23.71	90	55	76	49	33.8	0.13	3.276	134.39	99	105
33	Trans women	26	20.14	120	80	85	68	169.7	0.114	8.593	145.29	106	97.5
34	Trans women	30	31.25	125	85	87	26	87.5	0.347	9.552	135.26	109	96
35	Trans women	32	28.28	112	70	83	158	56.8	0.256	10.876	161.05	100	91
36	Trans women	29	20.34	120	70	79	74	136.1	0.231	9.674	220.88	93	102.8
37	Trans women	28	18.65	100	60	81	24	200.1	0.143	14.851	159.34	88	85.5
38	Trans women	29	29.26	110	80	92	54	40.4	5.935	6.848	240.16	109	102
39	Trans women	25	23.73	115	80	84	55	28.9	0.153	7.114	245.59	111	67.8
40	Trans women	37	28.18	120	80	125	47	16.3	0.186	9.104	175.8	91	115.5
41	Trans women	21	23.7	110	78	81	54	40.5	0.235	8.777	178.01	112	999
42	Trans women	19	19.43	113	72	87	43	31.6	0.606	6.594	227.65	96	999
43	Trans women	28	29.17	130	85	83	24	97	0.147	11.011	161.02	116	87
44	Trans women	29	27.31	120	80	80	24	32.5	0.183	12.702	173.87	95	999
45	Trans women	36	29.76	110	80	97	47	25.3	1.762	12.079	219.81	105	116.6
49	Cis women	38	37.34	110	80	79	31.1	62.6	0.22	7.216	148.69	81.2	92

50	Cis women	33	18	100	60	88	93.5	90.7	0.025	6.076	198.08	115.1	124.62
51	Cis women	32	27.92	100	60	82	175.3	58.5	0.25	10.045	236.01	107.3	79.86
53	Cis women	29	26.08	120	80	85	63.4	79.1	0.32	9.167	246.94	88.4	79.9
54	Cis women	34	21.38	999	999	89	11.2	115.2	0.09	8.542	227.45	88.3	88
55	Cis women	31	28.6	100	60	84	72.1	66.9	0.21	9.073	183.65	118.6	118
56	Cis women	38	33.33	110	70	89	113.3	29.9	0.15	12.722	149.75	104.9	115
57	Cis women	33	25.65	999	999	85	124.1	112	0.29	8.53	180.9	112	115.6
58	Cis women	31	26.67	100	70	89	46.8	5.7	0.17	8.997	166.71	98.6	99
59	Cis women	34	21.51	120	80	87	6.62	999	0.42	7.36	173.33	103.9	74.41
60	Cis women	39	23.44	100	80	74	576.8	72.2	0.1	11.877	166.19	96.3	110.7
61	Cis women	24	20.96	100	80	78	37.5	26.5	0.1	9.841	134.53	92.5	100.1
62	Cis women	40	28.6	100	60	93	72.2	45	0.31	5.047	210.87	120.2	170
63	Cis women	27	22.04	120	70	86	64.9	34.6	0.37	7.308	166.16	100.9	92.1
64	Cis women	39	28.06	120	90	84	221.2	38.3	0.28	8.683	248.1	103	113
65	Cis women	40	34.05	110	80	79	59.7	31.5	0.26	11.779	114.21	109.4	104.19
66	Cis women	24	24.97	120	80	83	63.4	54.1	0.33	5.228	157.65	101.8	106
68	Cis women	32	20.94	110	62	88	40	139.3	0.212	5.656	143.86	104	999
69	Cis women	26	20.42	110	75	78	114	55.7	0.453	8.472	136.52	105	999
72	Cis women	22	20.37	100	60	85	254	46.7	0.394	8.159	176.42	96	999
73	Cis women	22	26.7	100	78	91	42	61.2	0.216	9.032	258.73	107	999
74	Cis women	24	22.5	90	60	89	431	74.5	0.473	9.285	165.52	94	999
78	Cis women	22	24.51	95	75	84	104	37.1	0.68	9.07	201.72	113	999
79	Cis women	23	19.43	88	60	87	50	64.2	0.31	11.317	293.49	103	999
83	Cis women	35	26.37	100	78	85	110	67.8	0.498	6.551	178.05	110	999
85	Cis men	30	25.43	100	70	86	21.6	20.6	2.98	4.578	202.68	112.7	89.57
86	Cis men	34	26.25	120	80	95	16.9	24.2	3.33	8.938	192.98	112.6	118.8
87	Cis men	33	25.56	110	70	84	27.7	34	5.1	6.755	227.71	95.7	113
88	Cis men	31	25.95	120	80	74	24.9	40.5	5.21	7.824	262.63	103.5	123.7
89	Cis men	24	27.17	999	999	88	26	33.1	5.69	3.834	124.49	117.6	97.75
90	Cis men	27	32.53	130	90	88	35	26.3	4.71	5.436	219	110.3	131
91	Cis men	38	29.43	110	80	95	17.5	29.7	3.33	5.705	204.87	96.5	130.9
92	Cis men	36	32.25	130	80	90	16.1	23.2	3.62	6.715	224.75	109.2	147
93	Cis men	38	26.17	120	80	83	20.9	27.9	5.2	8.026	176.25	92.2	95.5
94	Cis men	18	20.69	110	70	88	35.9	34.8	7.16	4.75	181.57	110.8	140
95	Cis men	34	21.98	110	80	94	18.6	23.4	3.79	10.995	192.79	116.1	128.2
96	Cis men	35	28.41	120	80	90	14.1	26.5	3.99	6.615	243.89	133.2	121
97	Cis men	22	24.73	120	80	84	19.8	41.9	4.75	6.656	209.61	89.2	71
98	Cis men	22	21.13	110	70	92	10	42.2	7.03	5.589	162.58	108	128.1
99	Cis men	40	21.11	110	70	82	30.5	67.9	9.96	3.801	273.1	102.8	68.54
100	Cis men	28	25.68	100	70	82	15.1	31.4	5.21	6.099	135.38	94.9	130.2
101	Cis men	35	24.02	120	80	98	13.8	20.9	5.27	4.997	172.89	138.5	155.5
102	Cis men	32	29.56	120	80	94	36.6	15.9	4.03	6.482	244.83	112.5	100
103	Cis men	35	25.8	110	80	93	14.5	15.9	3.19	8.27	175.92	124.5	62.62

105	Cis men	21	22.78	999	999	93	20.4	34.6	5.46	10.8	235.57	109.1	100
106	Cis men	31	31.14	110	80	81	14	15.5	2.94	0.932	234.66	97.2	105.4
107	Cis men	25	20.68	110	70	86	34.3	60.3	8.49	0.753	195.58	99.9	97.2
112	Cis men	26	23.98	110	85	92	24	16.8	5.67	6.436	185.59	115	999
114	Cis men	20	21.35	115	70	96	24	33.7	6.62	9.71	197.66	108	999
119	Cis men	22	24.27	120	78	83	26	19.8	5.01	9.426	312.81	114	999

ID	Anticoagulant Protein C, %	Prothrombin activity, %	Thrombin time, s	Fibrinogen, mg/dL	Leukocytes, $x10^3/\mu L$	hs-CRP, mg/L
1	112	103	11.6	322	10.01	0.81
2	134	117	10.2	330	13.29	2.02
3	137	999	19.8	999	7.09	999
4	131	126	11.6	335	7.31	3.88
5	125	109	10.4	356	6.59	0.75
6	112	99	10.4	371	11.67	1.4
7	114	84	12.1	275	10.94	1.1
8	90	84	11.6	350	9.99	3.44
9	130	93	10.6	385	7.03	3.6
10	113	93	10.6	252	5.17	0.32
11	119	93	22.1	285	4.16	1.9
12	157	95	11.8	244	6.73	5.61
14	128	103	11.1	342	7.39	0.68
16	145	99	10.6	311	4.06	4.86
17	114	114	11.1	241	8.25	0.7
18	123	106	10.4	315	7.38	1.17
19	133	119	11.9	190	4.04	1.71
20	105	89	12	341	13.17	0.83
21	109	99	11.7	344	8.43	3.1
22	71	91	10.1	258	8.56	0.37
23	116	104	10.7	302	5.24	2.52
24	131	107	10.5	434	5.63	1.54
25	145	70	11.5	278	6.74	3.5
27	93	84	10.3	387	8.48	4.4
28	140	126	11.6	338	7.01	4.8
31	149	126	9.4	391	6.49	0.87
32	102	98	15.4	338	7.94	2
33	103	94	20.3	320	8.47	1.9
34	100	91	18.7	329	8.12	3.3
35	107	96	20.6	207	9.52	3.8
36	100	100	20.3	238	8.55	1.5
37	109	100	20.7	284	8.77	0.6
38	110	84	18.8	183	7.89	4
39	80	91	17.2	325	6.01	7.4
40	118	100	22.1	256	6.92	0.5
41	105	89	999	213	5.23	0.4
42	93	84	999	295	3.53	0.9
43	103	86	17.3	245	6.22	0.7
44	114	100	999	339	10.39	2.7
45	136	97	16.3	266	8.28	1
49	60	95	11.7	311	7.3	4.8

50	121	999	11.8	255	3.97	0.23
51	101	86	11	205	6.24	0.45
53	95	86	11.6	224	5.98	0.7
54	999	112	12.5	255	5.25	2.03
55	98	126	11	500	7.56	4.7
56	116	126	11.9	390	8.37	7.2
57	107	122	10.2	362	5.09	3.06
58	106	126	5.15	312	5.15	3.45
59	124	95	11.4	215	4.82	0.18
60	131	103	11.1	292	9.82	0.67
61	114	92	10.9	285	4.38	0.32
62	108	101	10.8	244	5.17	1.4
63	126	83	11.4	279	5.38	0.31
64	100	90	10.7	344	8.47	1.29
65	112	110	10.5	379	7.64	4.39
66	131	105	11.2	311	7.34	0.67
68	89	100	999	303	4.34	0.5
69	128	100	999	355	9.58	1.5
72	93	92	999	236	7.44	0.4
73	122	100	999	262	8.21	2.2
74	97	100	999	248	5.03	0.8
78	89	100	999	236	9.8	0.4
79	116	83	999	303	6.06	0.4
83	102	100	999	242	5.43	0.4
85	132	126	12.9	271	6.94	1.45
86	131	84	11.6	384	4.37	3.29
87	95	94	11.4	232	4.14	0.21
88	91	78	10.9	252	5.53	0.54
89	110	101	12.3	302	6.53	0.46
90	92	87	11.4	320	3.97	1.9
91	94	126	11.3	306	7.52	4.17
92	999	105	11.3	272	6.77	2.96
93	80	74	11.2	222	4.86	0.72
94	141	123	11.8	296	6.38	0.68
95	130	107	10.8	263	6.37	0.74
96	999	110	10.4	325	4.94	0.43
97	72	60	11.1	236	4.61	0
98	105	102	12.5	252	3.73	1.63
99	96	103	12.1	219	5.48	0.18
100	104	103	13.1	289	5.24	0.96
101	162	103	11.6	230	6.31	0.34
102	137	107	11.4	349	6.69	1.94
103	135	103	11.1	290	6.92	0.99

105	100	101	11	268	5.66	0.14
106	103	90	11	282	5.49	0.66
107	74	82	11.2	292	4.38	0.98
112	100	95	999	213	5.59	0.9
114	99	90	999	212	6.31	1
119	102	88	999	270	6.52	0.4
